# Oral Probiotics to Prevent Recurrent Vulvovaginal Infections During Pregnancy—Multicenter Double-Blind, Randomized, Placebo-Controlled Trial

**DOI:** 10.3390/nu17030460

**Published:** 2025-01-27

**Authors:** Zohar Nachum, Abeer Suleiman, Raul Colodner, Shlomo Battino, Malak Wattad, Olga Kuzmin, Enav Yefet

**Affiliations:** 1 Department of Obstetrics & Gynecology, Emek Medical Center, Afula 1834111, Israel; 2 Ruth and Bruce Rappaport Faculty of Medicine, Technion—Israel Institute of Technology, Haifa 3478403, Israel; 3 The Holy Family Medical Center, Nazareth 1641100, Israelvoyga2009@gmail.com (O.K.); 4 Microbiology Laboratory, Emek Medical Center, Afula 1834111, Israel; 5 Women’s Health Center, Clalit Health Services, Afula 1834111, Israel; battino@clalit.org.il; 6 Azrieli Faculty of Medicine, Bar-Ilan University, Safed 5290002, Israel; 7 Department of Obstetrics and Gynecology, Tzafon Medical Center, Poriya 1528001, Israel

**Keywords:** pregnancy, probiotics, vulvovaginal infections, bacterial vaginosis, abnormal vaginal flora, vulvovaginal candidiasis, secondary prevention

## Abstract

**Objective**: During pregnancy, vulvovaginal infections (VVIs), including abnormal vaginal flora (AVF), bacterial vaginosis (BV), and vulvovaginal candidiasis (VVC), are associated with serious complications and discomfort. We aimed to elucidate the effectiveness of oral probiotics in secondary prevention of VVIs in pregnant women. **Study design**: A multicenter prospective randomized, double-blind, placebo-controlled trial was conducted at three medical centers between 2016 and 2021. Women who complained of vaginal symptoms with positive smear for AVF/BV and/or candida were treated with antibiotics or an antimycotic agent, respectively. After confirmation of VVI eradiation by repeated vaginal smear, the women were divided into a research group, receiving two capsules/day of oral probiotic formula containing *Bifidobacterium bifidum*, *Bifidobacterium lactis*, *Lactobacillus* (*L.*) *acidophilus*, *L. paracasei*, *L. rhamnosus* and *Streptococcus thermophilus* (>6 × 10^9^ CFU/capsule), and a control group, receiving a placebo (two capsules/day) until delivery. At least once a month or following complaints, a vaginal smear was taken to assess vaginal microbiota. If VVIs were found, they were treated with antibiotics/antimycotics, and eradication was assessed by a repeated vaginal smear. Lactobacilli vaginal colonization, including the specific strains from the probiotic capsules, were detected using the matrix-assisted laser desorption/ionization time-of-flight mass spectrometry (MALDI TOF-MS). The primary outcome was the rate of women who developed VVI during the study period until delivery. **Results**: Twenty-three and twenty-four women were analyzed in the probiotic and placebo cohorts, respectively. There was no difference in the rate of any VVI between the probiotic and placebo cohorts (16 (67%) versus 11 (48%), respectively; *p* = 0.19), time until first infection or pregnancy outcomes. The lactobacilli strains that colonized the vagina were similar at baseline and following probiotic or placebo administration. No woman was detected with vaginal colonization of the strains from the capsule, although the probiotics were taken for about 4 months. **Conclusions**: The oral probiotic product tested in this study did not reduce the recurrence rate of VVIs in pregnant women following eradication.

## 1. Introduction

Vulvovaginal infections (VVIs) represent a prevalent medical concern. The most common VVI is bacterial vaginosis (BV) or, its milder form, abnormal vaginal flora (AVF). These conditions are characterized by the replacement of the normal lactobacilli-dominated vaginal microbiota with anaerobic bacteria [1]. The second most frequent VVI is caused by *Candida* species, with *Candida albicans* being the primary agent responsible for vulvovaginal candidiasis (VVC) [2].

During pregnancy, VVIs are associated with significant maternal and neonatal complications. BV, which affects approximately 30% of pregnant women [3], has been linked to postabortal pelvic inflammatory disease, abnormal cervical cytology, premature rupture of membranes, preterm labor and delivery, chorioamnionitis, and post-cesarean endometritis [4,5]. VVC is also more prevalent during pregnancy, affecting around 20% of pregnant women [6]. Maternal VVC in the third trimester has been associated with vertical transmission of yeast infections, leading to oral thrush and diaper dermatitis in infants during their first year of life [7].

For BV/AVF in pregnancy, antibiotic therapy is the treatment of choice and has been shown to reduce the rates of late miscarriage and preterm delivery [8,9,10,11]. Similarly, vaginal antimycotic treatment is the preferred intervention for VVC. However, VVC recurrence is more frequent during pregnancy, and therapeutic response is diminished, likely due to the hormonal and immunological changes associated with gestation [2,3,12]. Furthermore, antibiotic and antimycotic treatments do not consistently result in the re-colonization of the vagina with normal microbiota, which may contribute to the recurrence of VVIs.

To date, oral products aimed at secondary prevention of VVIs following antibiotic or antimycotic treatment during pregnancy have not been thoroughly investigated and are not currently recommended.

Lactobacilli are the dominant microorganisms in normal vaginal microbiota and play a critical role in infection prevention by producing antimicrobial compounds such as hydrogen peroxide, lactic acid, and bacteriocin-like substances. Additionally, their ability to adhere to epithelial surfaces and compete for adhesion sites enhances their protective function [13]. Consequently, their use has been proposed as a preventive and therapeutic strategy for VVIs. Several studies in non-pregnant women have yielded promising results [14,15,16,17,18,19].

The gut microbiota has co-evolved with its human hosts and has become an integral component of the human body. Although the gut microbiota is dynamic in nature, it performs fundamental functions within the immunological, metabolic, structural, and neurological domains of human physiology. Furthermore, the gut microbiota exerts a significant influence on both the physical and mental well-being of individuals [20]. When the normal vaginal microbiota is disturbed by the depletion of lactobacilli, the gut may function as a reservoir for lactobacilli, which then colonize the vagina [21,22]. In pregnancy, the impact of oral probiotic supplements on VVI incidence has been explored [23]; however, no study has specifically evaluated the role of probiotics in secondary prevention of VVIs after their eradication. The oral route for probiotic administration during pregnancy should be explored due to several considerations: oral administration may be preferred during pregnancy due to the physiological increase in vaginal secretions, which can lead to patient discomfort and potentially affect the action of vaginally administered probiotics through mechanical washing; co-administration of vaginal pregnancy-related medications such as hormonal supplements can also cause patient discomfort associated with the vaginal route; and it was hypothesized that the gut may function as a reservoir for lactobacilli that colonize the vagina [21,22].

The present study aimed to evaluate the efficacy of oral probiotic supplementation for the secondary prevention of VVIs in pregnant women following the resolution of symptomatic infections. To this end, we used an oral probiotic supplement formula containing *Bifidobacterium bifidum Bb-06*, *Bifidobacterium lactis Bi-07*, *Lactobacillus* (*L.*) *acidophilus La-14*, *Lacticaseibacillus paracasei Lpc-37*, *Lacticaseibacillus rhamnosus Lr-32*, and *Streptococcus thermophilus St-21*. All these bacteria were shown to be safe during pregnancy [23,24] and to either colonize the normal vaginal microbiota [23,25,26] and/or be part of a probiotic formula that improved vaginal dysbiosis [27,28,29,30].

## 2. Material and Methods

A multicenter, double-blind, randomized, placebo-controlled trial was conducted between November 2016 and January 2021 in Israel at three university-affiliated sites: Emek Medical Center in Afula, the Holy Family Medical Center in Nazareth, and the Women’s Health Center of Clalit Health Services in Afula. This study was authorized by the review boards of the participating centers (ClinicalTrials.gov identifier: NCT02795845). The participants provided written informed consent.

Pregnant women above 18 years old up to their 30th week of gestation were tested for VVI due to vulvovaginal symptoms (e.g., vaginal discharge, pruritus, burning sensation, dryness, and erythema). Since BV/AVF and VVC share common pathogenesis, we combined them into a VVI group with separate randomization for each infection.

The presence of BV/AVF and VVC was assessed by taking a vaginal smear with a sterile transport swab. The Nugent score was used for AVF/BV diagnosis as previously described [31,32]. A Nugent score >3 was considered positive for AVF/BV. *Candida* infection was assessed with direct microscopy. Women were treated with antibiotics for BV/AVF. The treatment regimen was either oral metronidazole 500 mg twice daily or clindamycin 300 mg twice daily for seven days, or both if eradication was not achieved (in repeated vaginal smear) after one treatment cycle. VVC was treated with a vaginal tab of clotrimazole 500 mg twice a week for one week. If eradication was not achieved, the women were given the same treatment for an additional week. Eradication was verified by repeated vaginal smear. Women with normal vaginal flora (Nugent score < 4 and no *Candida*) in a smear following treatment(s) were recruited.

The exclusion criteria included failure to eradicate BV/AFV/VVC after two treatment cycles, preterm premature rupture of the membranes at enrollment, immunocompromised women (e.g., autoimmune diseases treated medically), *Trichomonas* infection at enrollment, and allergy to soy or fish (since capsules used in this study were manufactured in the same line as soy and fish). Women who took probiotic supplements and refused to discontinue treatment were also excluded.

### 2.1. Randomization and Masking

Randomization was conducted separately for women whose primary infection was BV/AVF versus VVC. In cases where both infections were present, randomization was performed according to the BV/AVF group. Participating women were randomly assigned (1:1) to treatment groups using a computer-generated randomization sequence with a block size of four. The randomization code was stored in a closed study box in sealed opaque envelopes until the study physicians assigned the intervention. The envelopes were marked with sequential numbers, and each envelope contained a code for one of the study groups. Participating women, study investigators, and laboratory personnel were blinded to the study allocation.

### 2.2. Interventions

Women were allocated to receive one capsule twice daily until the delivery of either an oral probiotic formula or a placebo. The probiotic formula contained *Bifidobacterium bifidum Bb-06*, *Bifidobacterium lactis Bi-07*, *Lactobacillus* (*L.*) *acidophilus La-14*, *Lacticaseibacillus paracasei Lpc-37*, *Lacticaseibacillus rhamnosus Lr-32*, and *Streptococcus thermophilus St-21* (>6 × 10^9^ CFU/capsule; manufacturer: Danisco USA Inc., Thomson, IL, USA, batch number 61002442). The placebo capsules were identical in appearance to the probiotic capsules and packaged in the same containers. Before trial initiation, we cultured the lactobacilli from a sample of the probiotic capsules to ensure their viability and detectability using our culture techniques. Monthly evaluations and assessments in case of symptoms consistent with VVI included:

(1) Clinical evaluation of VVI signs and symptoms, including subjective complaints of vaginal discharge, pruritus, burning sensation, dryness, and erythema, as well as objective evaluation of vaginal discharge and erythema. Each parameter was rated as absent, mild, moderate, or severe.
(2) Vaginal smear for direct microscopy to evaluate the presence of AVF/BV (defined as Nugent score above 3) and *Candida*.
(3) Patient interviews for possible adverse effects.
(4) Verification of adherence to instructions not to consume other probiotic supplements, confirmed at each visit by direct questioning.
(5) Compliance assessment by counting the remaining capsules returned by patients at each visit.

In cases of VVI, the women were treated with antibiotics for BV/AVF (either oral metronidazole 500 mg twice daily or clindamycin 300 mg twice daily for seven days, or both if eradication was not achieved after one treatment) or vaginal clotrimazole 500 mg twice weekly for one week for VVC. An additional week of treatment was given if eradication was not achieved. Eradication was assessed by repeated vaginal smear. Throughout this period, the study products were continued until delivery.

### 2.3. Identifying Lactobacilli Strains Including the Specific Strains of the Probiotic Product

Before initiating treatment with either probiotic capsules or placebo, and after 1–2 months and 3–4 months of treatment, vaginal samples were obtained for bacterial culture. A semi-quantitative assessment of vaginal lactobacilli was performed using selective culture plates for this strain, and the specific lactobacilli from the probiotic capsules were sought in each group. To determine the presence of lactobacilli in vaginal specimens and enumerate the number of colony-forming units (CFUs), serial dilutions were plated on lactobacillus agar according to De Man, Rogosa, and Sharpe (Rogosa Agar) [33]. Confirmation of lactobacilli growth was made by Gram staining (Gram-positive rods). The pattern of bacterial growth was used for semi-quantitative interpretation on a scale from 0 (no vaginal colonization) to 4 (substantial colonization).

### 2.4. Identifying the Specific Lactobacilli of the Probiotic Product

For this study, the lactobacilli from the probiotic capsules were isolated, and matrix-assisted laser desorption/ionization time-of-flight mass spectrometry (MALDI-TOF-MS) was used to create a specific and highly accurate profile for each strain. In a preliminary step, the specific strains from the probiotic capsules were cultured in the same conditions (culture media, temperature and time) of the future clinical samples. Following the instructions of MALDI-TOF’s manufacturer (Bruker Daltonics, Bremen, Germany), a total of 20 replicates for each strain were used to build a specific in-house profile, which was introduced to the instrument database. The ability of the system to correctly identify the capsule lactobacilli was locally validated against the American Type Culture Collection (ATCC) and wild strains from the same species. An identification score higher than 2.2 was strongly correlated with capsule-originated colonies, given the culture conditions were highly maintained [34]. In a dendrogram built with all those profiles, the capsule-originated isolates were all in a separated cluster. Similar methods have been used by other authors to reach sub-species level of identification [35,36].

The cultures described in the previous section were used to identify the lactobacilli strains, including the specific strains from the probiotic capsules. Bacteria from 2–3 independent colonies were identified using MALDI-TOF-MS, as previously described [37,38].

Briefly, bacteria isolated from Rogosa agar were seeded on the MALDI-TOF-MS wells and acquired an electrical charge by being mixed with a special matrix. The mixture was then applied to a metal plate. A pulsed laser irradiated the sample, triggering ablation and desorption of the sample and matrix material. Finally, the analyte molecules were ionized by being protonated or deprotonated in the hot plume of ablated gases, and could then be accelerated into a mass spectrometer for analysis [39].

This database was used to identify those bacteria (dendrogram) in the vaginal samples obtained from all study groups. The results of the MALDI-TOF-MS were kept in the microbiology laboratory and were disclosed to the investigators only after study completion.

### 2.5. Study Endpoints

The primary endpoint was the rate of women who developed any VVI (BV/AVF/VVC) during the study period until delivery. Secondary endpoints included the rates of women who developed AVF/BV, VVC, or urinary tract infection (UTI) during the study period. Additional outcomes were the duration from study initiation to the first episode of VVI (either AVF/BV or VVC), the rate of VVI at each monthly study visit until delivery, and data regarding treatment results for VVIs. Obstetrical outcomes assessed included the mode of delivery, obstetrical complications and pregnancy-related disorders (e.g., gestational diabetes mellitus, pre-eclampsia), preterm delivery, being small for gestational age, preterm premature rupture of membranes, chorioamnionitis, and postpartum endometritis. Neonatal outcomes included Apgar scores at 1 and 5 min after birth, neonatal sepsis, and complications such as acute respiratory distress syndrome, intraventricular hemorrhage, and admission to the neonatal intensive care unit. Maternal adverse effects were also documented.

### 2.6. Statistical Analysis

The recurrence rate of BV/AVF following antibiotic treatment was approximately 70% and 35% in the placebo and probiotic groups, respectively [15]. For VVC in non-pregnant women, the recurrence rates following antimycotic treatment were 12.5% and 2.5% in the placebo and probiotic groups, respectively [18]. Given the higher prevalence and recurrence rates of VVC in pregnancy and symptomatic women [2,12], we estimated a recurrence rate of 25% in the placebo group compared to 5% in the treatment group. Assuming a reduction from 60% to 27% in the appearance of VVIs in the study group compared to control, a total of 68 women were required (80% power, 5% two-sided alpha).

Inter-cohort baseline characteristics and outcomes were compared using Student’s *t*-test (or the Wilcoxon two-sample test) for continuous variables and χ^2^ (or Fisher’s exact test) for categorical variables. We evaluated the time from enrollment until the first VVI episode in each cohort using the Kaplan–Meier curve in weeks. A log-rank test was performed to compare the groups’ survival curves.

Statistical analyses were carried out with SAS version 9.4 (SAS Institute, Cary, NC, USA). Significance was set at a *p* value < 0.05.

## 3. Results

**Figure 1:**
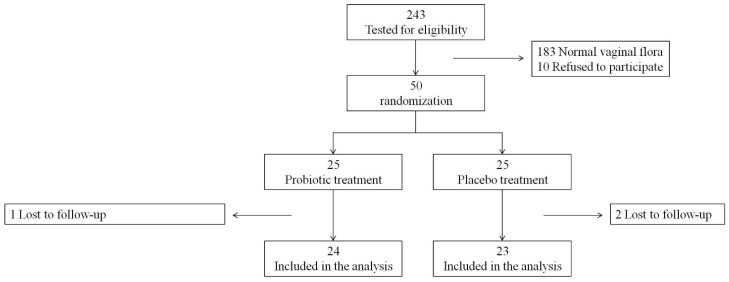
Patient flow chart.

A patients flow chart is presented in Figure 1. During the study period (more than 4 years), only 60 women out of the 243 who were screened were eligible to participate in this study. Of them, 50 women (83%) who were recruited accounted for 74% of the total sample size required. This study was terminated before the calculated sample size was reached since the rate of women who were eligible to participate was lower than expected and the financial resources to complete the study were exhausted.

Overall, 24 and 23 women were allocated to the probiotic and placebo groups, respectively, and were included in the analysis. 

The patients’ characteristics were comparable between the groups except from earlier gestational week at the beginning of the study treatment in the probiotic group (Table 1). There were no women that had an allergy to metronidazole, clindamycin or clotrimazole.

Compliance with the study products’ administration was high and comparable in both groups (81 ± 27% in the probiotic group versus 81 ± 24% in the placebo group; *p* = 0.89).

**Figure 2:**
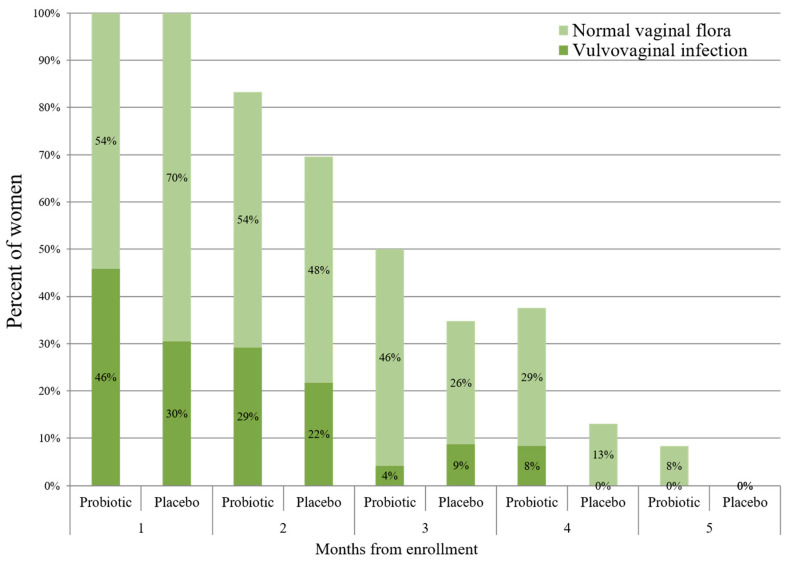
The rate of vulvovaginal infections (VVIs) throughout this study in the probiotic (N = 24) and placebo (N = 23) groups. The study participants were invited for a vaginal swab once in every month until delivery. Repeated vaginal swabs for verifying VVI eradication are not presented. *p* > 0.05 for all the time points.

The mean study duration was 15.4 ± 5.6 and 12.4 ± 4.2 weeks in the probiotic and placebo groups, respectively (*p* = 0.07). There was no statistically significant difference between the groups in the rate of VVI, AVF/BV, VVC, or the rate of UTI (Table 2). Time until first infection appearance was comparable between the groups (Table 2 and Figure 2).

**Figure 3:**
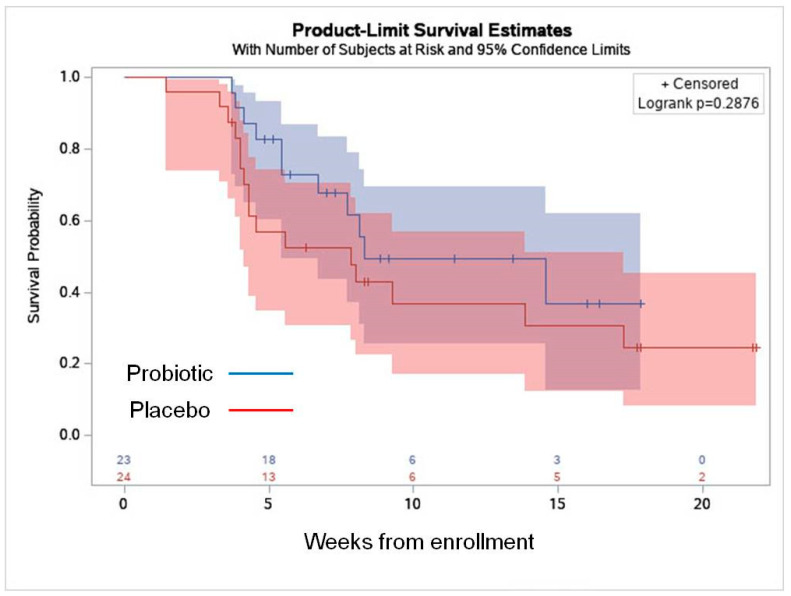
Kaplan–Meier survival curve representing the time from enrollment to the first vulvovaginal infection.

The rate of VVI at each visit is presented in Figure 3. There was no statistical difference between the probiotic and placebo groups at any of the time points (*p* > 0.05 for all the comparisons).

Pregnancy outcomes were comparable between the groups, including the mode of delivery, the rate of chorioamnionitis, endometritis and intrapartum fever (Table 2).

Subjective symptoms and objective vaginal examination at baseline, one month following treatment with the study products and at the last visit of this study are presented in Table 3. There was no difference between the groups in all the time points, except for pruritus, which was reported less in the probiotic group at the last visit (*p* = 0.03, Table 3).

Data regarding the treatment results of the VVI events are presented in Table 4. In the placebo group, all the VVI events (N = 4) were resolved after one or two treatment cycles. In the probiotic group, 11/13 (85%) VVI events were resolved (*p* = 1).

**Figure 4:**
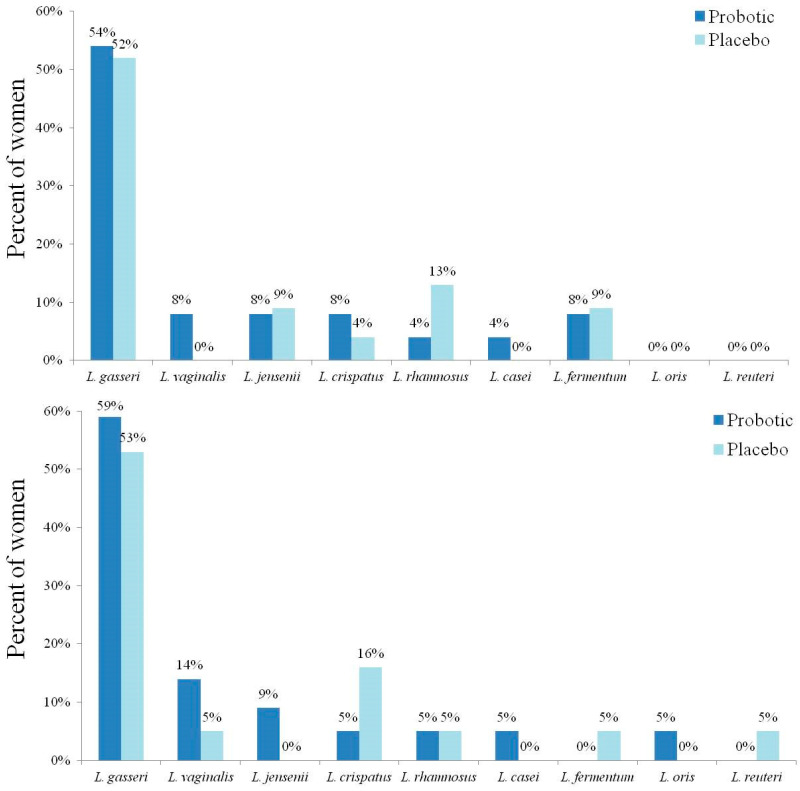
Percentage of vaginal lactobacilli (L.) strains from the study participants according to the study groups at baseline (lower panel; N = 22 in the probiotic group and N = 19 in the placebo group) and in the last vaginal culture (upper panel; N = 24 in the probiotic group and N = 23 in the placebo group).

The assessment of vaginal lactobacilli colonization demonstrated a similar growth in both groups (Table 2). The dominant lactobacilli identified using MALDI-TOF-MS at baseline and at the last available vaginal specimens in each group are presented in Figure 4. The most dominant strain was *L. gasseri* in both groups (Figure 4). No woman was found to have vaginal colonization of the specific bacterial strains of the probiotic supplements in all the available specimens that were cultured. Comparison of the bacterial composition between baseline and the last culture for each group was not statistically significant (*p* > 0.05).

At baseline, in four women in each group, two L. strains were identified in culture. At the last culture, in three and four women, two L. strains were identified in the probiotic and placebo groups, respectively. The strains are listed in all the appropriate places. Comparison of the bacterial composition between baseline and the last culture for each group was not statistically significant (*p* > 0.05 in all the comparisons).

Four (17%) and two (9%) women reported gastrointestinal adverse effects in the probiotic and placebo groups, respectively (*p* = 0.67).

Neonatal outcomes were similar between the groups (Table 5).

Since fewer women were enrolled than the calculated sample size, we performed a sensitivity analysis assuming that the rest of the sample size was recruited and all had the primary outcome (VVI) in the placebo group and did not have the primary outcome in the probiotic group. Following this assumption, there was still no statistically significant difference between the groups (16/34 (47%) and 22/34 (65%) in the probiotic and placebo groups, respectively, *p* = 0.14).

## 4. Discussion

In the present study, we investigated the effect of oral probiotic administration in preventing VVIs in pregnant women as a secondary prevention strategy after eradication of VVI with antibiotic or antimycotic treatment. We found no statistically significant difference in the rate of VVI between the probiotic and placebo groups, nor in any other parameter related to maternal, pregnancy, and neonatal outcomes. The lactobacilli colonizing the vagina were similar at baseline and following probiotic or placebo administration. Notably, the specific lactobacilli strains from the probiotic capsules were not detected in the vagina despite the probiotics being taken for an average of about 4 months.

The effect of probiotics on eradication of BV and VVC has been studied previously [23]. Most studies were conducted in non-pregnant women [23] and used vaginal probiotic treatments [40,41,42,43]. Only a few studies administered oral probiotic supplements [44,45]. The results are conflicting, with some studies demonstrating an effect on the vaginal microbiota and lower recurrence rates of VVI, while others showed no beneficial effect of probiotics on the vaginal microbiota [23]. Since these studies used a combination of antibiotics or antimycotic treatment with probiotics without confirming eradication prior to the initiation of study interventions, the effect of probiotics on secondary prevention could not be assessed. In pregnancy, studies examining the effect of probiotics on vaginal microbiota included either women with VVI or a mixed population of women with VVI or normal vaginal microbiota [46,47,48]. Secondary prevention was not assessed. This study provided important data due to the fact that orally administered probiotics demonstrated vaginal effects in non-pregnant individuals [23] and the objective of this study had not been previously examined using an appropriate methodology.

To our knowledge, this is the first study investigating the effect of oral probiotics on secondary prevention of VVI during pregnancy after VVI eradication was confirmed. This issue is important due to several aspects: firstly, since VVIs are associated with obstetric complications, rapid eradication is mandatory. Secondly, if eradication is not achieved, which may occur in up to 50% of women [11,49], a second line of antibiotic or another course of antimycotic treatment should be used, and this was not evaluated in previous studies. Thirdly, women in whom eradication was successful might respond differently to probiotic treatment than women with persistent VVI. However, since eradication was not evaluated in previous studies, their study populations comprised women with and without VVI at the beginning of the studies, which might lead to bias. This might explain why some studies demonstrated a positive effect while others did not [23].

In this study, *L. gasseri* was the dominant lactobacilli in the vagina. Interestingly, longitudinal analysis of the vaginal microbiota in pregnancy demonstrated that *L. gasseri* incurred a ten-fold increased risk of conversion to abnormal vaginal microbiota relative to non-*L. gasseri* vaginal microbiota [50]. In two studies, the vaginal probiotic mixture that included *L. gasseri* did not improve VVI cure rates or alleviate recurrence [40,42]. These observations may support the hypothesis that different lactobacilli strains might lead to different protection levels against VVIs. Future studies should focus on examining this hypothesis.

Fewer women reported pruritus in the probiotic group at the last visit of this study. Since this was the only difference between the groups, further investigation is needed to assess whether it is a true effect or potentially related to chance.

In the probiotic group, there was a trend for more cases of NICU admissions. This is probably attributed to the higher rates of twins in the probiotic group, as three pairs of twins (six babies) were admitted to the NICU unit.

The strengths of this study were its multicenter, randomized, placebo-controlled design, high compliance rate, use of methods to detect specific bacterial strains from the capsule, and investigation of secondary prevention of VVI in pregnancy as a primary outcome in a homogeneous population after eradication was confirmed.

The limitations of this study were that the calculated sample size was not reached; there was an insufficient sample size to evaluate pregnancy outcomes and to evaluate separately the effect on BV/AVF and VVC; and the results are specific to the probiotic product used in this study. This product was chosen because it contained bacterial species that have beneficial effects on the vaginal microbiome and was commercially available, allowing for easy purchase if positive effects were demonstrated. The fact that we did not have information regarding the recurrence of VVI in study participants before pregnancy and that we did not analyze stool samples to guarantee adherence to the treatment, are also limitations of this study.

## 5. Conclusions

The oral probiotic product tested in this study did not reduce the recurrence rate of VVIs in pregnant women following eradication.

## Figures and Tables

**Table 1:** Baseline patients’ characteristics.

Characteristics	Probiotic N = 24	Placebo N = 23	* p * Value
Maternal age, years	30.5 (4.9) [31, 28–33]	30.4 (5.1) [30, 27–32]	0.92
Pre-pregnancy BMI, kg/M^2^	23.6 (4.4) [24.4, 20.1–26.1]	25.3 (5.4) [24.2, 21.5–26.6]	0.52
BMI at enrolment, kg/M^2^	25.2 (4.4) [24.9, 22.2–27.6]	27.7 (5.6) [26.6, 23.1–30.5]	0.09
Weight at enrolment, kg	69.0 (12.9) [67.5, 58.5–78.5]	72.3 (15.5) [68, 60–83]	0.43
Pregnancy number	2.5 (1.2) [2.5, 1.5–3.0]	2.8 (1.5) [3.0, 1.0–4.0]	0.51
No. of previous births	1.0 (0.9) [1.0, 0–1.5]	1.4 (1.3) [1.0, 0–2.0]	0.31
Risk factor for preterm birth	13 (54%)	9 (39%)	0.3
Num. of previous preterm births	0.3 (0.6) [0, 0–0]	0.2 (0.7) [0, 0–0]	0.74
Gestational week at beginning of study treatments	22.9 (5.0) [22.9, 20.0–27.1]	26.3 (4.6) [25.6, 22.9–30.0]	0.02
Cigarette smoking before pregnancy	6 (25%)	7 (30%)	0.68
Current cigarette smoking	3 (13%)	1 (4%)	0.61
Marital status: married	24 (100%)	21 (91%)	0.23
Years of education	14.2 (2.6) [12.5, 12.0–16.5]	14.3 (2.5) [14.0, 12.0–16.0]	0.82
Probiotic food in the diet	3 (13%)	0 (0%)	0.23
Infection in first vaginal swab: BV	15 (63%)	11 (48%)	0.58
Candida	8 (33%)	10 (43%)	
BV + candida	1 (4%)	2 (9%)	
Vaginal lactobacilli colonization *	18 (82%)	13 (68%)	0.47
Regular antibiotic use	0	0	-
Antimycotic use during pregnancy	1 (4%)	1 (4%)	1
UTI during pregnancy	5 (21%)	2 (9%)	0.42
Num. of UTI events during pregnancy	0.2 (0.4) [0, 0–0]	0.1 (0.5) [0, 0–0]	0.29
Multiple gestation	4 (17%)	1 (4%)	0.35
Gestational diabetes mellitus	3 (13%)	3 (13%)	1
Pre-eclampsia/gestational hypertension	1 (4%)	1 (4%)	1

Values are presented as mean (standard deviation) [median, IQR) or number (percent).* Refers to score ≥ 1 at semi-quantitative assessment of vaginal lactobacilli colonization (N = 22 and N = 19 in the probiotic and placebo groups, respectively). Missing: Vaginal pH and BV sense—2 Abbreviations: BMI, body mass index; BV, bacterial vaginosis; UTI, urinary tract infection.

**Table 2:** Study endpoints—maternal outcomes.

Outcomes	Probiotic N = 24	Placebo N = 23	* p * Value
VVI *	16 (67%)	11 (48%)	0.19
VVC	11 (46%)	9 (39%)	0.64
BV/AVF	11 (46%)	10 (43%)	0.87
Time until first infection (weeks)	6.2 (4.2) [4.3, 3.9–7.9]	6.6 (3.1) [5.4, 4.1–8.1]	0.40
Gestational week at first vaginal infection **	29.6 (5.5) [29.0, 26.5–34.0]	32.5 (3.1) [33.0, 30.0–34.0]	0.17
Recurrent event of VVI	1 (4%)	2 (9%)	0.61
Vaginal lactobacilli colonization ***	20 (83%)	16 (70%)	0.27
Delivery week	38.3 (2.1) [38.6, 37.8–39.6]	38.6 (1.4) [38.4, 37.9–39.9]	0.97
Preterm delivery	2 (8%)	1 (4%)	1
Delivery mode: vaginal	13 (54%)	15 (65%)	0.81
Vacuum	2 (8%)	2 (9%)	
Cesarean delivery	9 (38%)	6 (26%)	
PPROM	1 (4%)	1 (4%)	1
Chorioamnionitis	0	0	
Intrapartum fever	0	1 (4%)	0.49
Endometritis	0	0	
Study duration (weeks)	15.4 (5.6) [15.5, 10.8–18.6]	12.4 (4.2) [12.4, 9.4–16.0]	0.07

Values are presented as mean (standard deviation) [median, IQR) or number (percent). One woman had a late abortion. Neonatal outcomes are missing in this case. * At least one episode. ** Refers to women who had a vaginal infection during the study. *** Refers to score ≥ 1 at semi-quantitative assessment of vaginal lactobacilli colonization in the last culture available. Abbreviations: AVF, abnormal vaginal flora; BV, bacterial vaginosis; PPROM, preterm premature rupture of membrane; UTI, urinary tract infection; VVC, vulvovaginal candidiasis; VVI, vulvovaginal infection.

**Table 3:** Vulvovaginal symptoms and signs.

	Baseline			One Month			End of the Study		
Outcomes	Probiotic N = 24	Placebo N = 23	*p* Value	Probiotic N = 24	Placebo N = 23	*p* Value	Probiotic N = 24	Placebo N = 23	*p* Value
Subjective vulvovaginal symptoms	24 (100%)	23 (100%)		21 (88%)	20 (87%)	1	17 (71%)	20 (87%)	0.29
Vaginal discharge severity: None	0	0	0.12	3 (13%)	5 (22%)	0.38	7 (29%)	3 (13%)	0.31
Mild	8 (33%)	7 (30%)		8 (33%)	6 (26%)		9 (38%)	8 (35%)	
Moderate	15 (63%)	10 (43%)		12 (50%)	8 (35%)		8 (33%)	10 (43%)	
Severe	1 (4%)	6 (26%)		1 (4%)	4 (17%)		0 (0%)	2 (9%)	
Pruritus	12 (50%)	15 (65%)	0.29	8 (33%)	9 (39%)	0.68	3 (13%)	9 (39%)	0.03
Burning sensation	8 (33%)	6 (26%)	0.59	3 (13%)	2 (9%)	1	2 (8%)	3 (13%)	0.67
Dryness	3 (13%)	1 (4%)	0.61	1 (4%)	1 (4%)	1	3 (13%)	1 (4%)	0.61
Erythema	5 (21%)	1 (4%)	0.19	2 (8%)	1 (4%)	1	1 (4%)	1 (4%)	1
Objective vaginal discharge	23 (96%)	20 (87%)	0.35	20 (83%)	16 (70%)	0.27	15 (63%)	15 (65%)	0.85
Objective vulvovaginal erythema	0	0	-	2 (8%)	0 (0%)	0.49	0	0	-

Positive finding was considered at least mild severity.

**Table 4:** Response to treatment of VVI events.

	Eradication	Probiotic	Placebo
VVI	After one treatment cycle	7	3
	After two treatment cycles	4	1
	No eradication after two treatment cycles	2	0
	Unknown *	4	9
AVF/BV	After one treatment cycle	7	4
	After two treatment cycles	2	1
	No eradication after two treatment cycles	2	0
	Unknown **	1	8
VVC	After one treatment cycle	5	4
	After two treatment cycles	2	0
	No eradication after two treatment cycles	1	0
	Unknown **	3	6

Number of VVIs (AVF/BV and VVC) is presented. One and two women in the probiotic and placebo groups, respectively, had two VVI events and all of them are listed. Co-infection with both AVF/BV and VVC is listed in all the appropriate rows. In AVF/BV and VVC, eradication was considered the resolution of the infection that the treatment was targeting. In VVI, eradication was considered a complete resolution to normal vaginal microbiota. * In two and four women in the probiotic and placebo groups, respectively, VVI was not resolved after one treatment cycle and delivery took place before additional treatment or before a repeated vaginal smear was taken. ** Delivered before treatment or before repeated vaginal smear was taken. Abbreviations: AVF, abnormal vaginal flora; BV, bacterial vaginosis; VVC, vulvovaginal candidiasis; VVI, vulvovaginal infection.

**Table 5:** Study endpoints—neonatal outcomes.

Outcomes	Probiotic N = 28	Placebo N = 24	* p * Value
SGA	4 (14%)	1 (4%)	0.36
Apgar score at 1 min < 7	0	2 (8%)	0.21
Apgar score at 5 min < 7	0	0	
NICU admission	7 (25%)	1 (4%)	0.06
Neonatal sepsis	0	0	
Neonatal RDS	0	0	
IVH	1 (4%)	0 (0%)	1

Values are presented as a number (percent). Abbreviations: IVH, intraventricular hemorrhage; NICU, neonatal intensive care unit; RDS, respiratory distress syndrome; SGA, small for gestational age.

## Data Availability

The data from this study is available from the corresponding author upon a reasonable request and following approval of the institutional review board.
